# Quantification of fibrinogen-to-pre-albumin ratio provides an integrating parameter for differential diagnosis and risk stratification of early-stage colorectal cancer

**DOI:** 10.1186/s12935-022-02532-y

**Published:** 2022-03-27

**Authors:** Hou-Qun Ying, Wei Chen, Cui-Fen Xiong, Yuanyuan Wang, Xiao-Juan Li, Xue-Xin Cheng

**Affiliations:** 1 Department of Nuclear Medicine, Jiangxi Province Key Laboratory of Laboratory Medicine, The Second Affiliated Hospital of Nanchang University, Nanchang, 330006 Jiangxi China; 2 Jiangxi Medical College, Nanchang University, Nanchang, 330006 People’s Republic of China; 3 Department of Laboratory Medicine, Jiangxi Province Key Laboratory of Laboratory Medicine, The Second Affiliated Hospital of Nanchang University, Nanchang, 330006 Jiangxi China; 4 Department of Clinical Laboratory, Kunming Children’s Hospital, Kunming, 650103 Yunnan China; 5 Biological Resource Center, The Second Affiliated Hospital of Nanchang University, No.1 of Minde Road, Nanchang, 330006 Jiangxi China; 6 School of Public Health, Nanchang University, Nanchang, 330006 People’s Republic of China; 7 Jiangxi Provincial Key Laboratory of Preventive Medicine, Nanchang University, Nanchang, 330006 People’s Republic of China

**Keywords:** Pre-albumin to fibrinogen ratio, Colorectal cancer, High-relapse risk, Early-diagnosis, Inflammation

## Abstract

**Background:**

Circulating fibrinogen to pre-albumin ratio (FPR) and albumin to fibrinogen ratio (AFR) are effective factors for predicting the prognosis of colorectal cancer (CRC). However, the role of these two ratios in diagnosing early-stage CRC and identifying the stage II CRC subgroup with high relapse risk remains unknown. This study aimed to assess the potential of FPR and AFR in differential diagnosis and risk stratification of early-stage CRC.

**Methods:**

A discovery (694 and 512 patients with benign colorectal polyps and stage I–II CRC, respectively) and validation (201 benign colorectal polyps cases and 202 stage I–II CRC individuals) cohorts were enrolled in this study. Receiver operating characteristic curve (ROC), Kaplan–Meier curve, and time-dependent ROC were used to evaluate the diagnostic efficacy of AFR and FPR in the two cohorts and overall population, and the discriminating role of FPR in identifying clinical high-relapse risk patients in comparison with common clinical characteristics in stage II CRC patients.

**Results:**

The area under the curve (AUC) of the preoperative circulating FPR was higher than that of AFR in the diagnosis of stage I–II CRC from colorectal adenomas and benign colorectal polyps in the discovery and validation cohorts and overall population. Carcinoembryonic antigen (CEA) combined with FPR could effectively discriminate early-stage CRC from colorectal adenomas or benign polyps. Preoperative FPR could effectively distinguish stage II subgroups with high and low relapse risk. It was superior to common clinical characteristics in identifying high-risk surgical patients who could benefit from adjuvant chemotherapy (CT) [time-dependent AUC: 0.637 vs. 0.511, *p* < 0.001 for predicting recurrence-free survival (RFS); 0.719 vs. 0.501, *p* < 0.001 for predicting overall survival (OS)]. Furthermore, CT treated stage II patients with FPR > 20 had the highest recurrence (31.16%) and death rates (21.88%), with similar highest recurrence (30.70%) and death (26.82%) rates found in non-CT-treated patients with FPR > 20. Stage II CRC patients with 20 ≥ FPR > 15 could significantly benefit from postoperative CT, as the recurrence (33.30%) and death (35.71%) rates within non-CT treated patients were approximately five times higher than those of the CT-treated cases (6.77% and 7.41% for the recurrence and death rates, respectively). No significant difference in recurrence rate was observed between L-FPR (≤ 15) patients with (10.00%) or without CT (9.76%), indicating that these patients might not require to receive adjuvant CT after curative resection.

**Conclusions:**

Preoperative FPR combined with CEA is superior to common tumor biomarkers, FPR, or AFR in distinguishing early-stage CRC from benign colorectal polyps. Circulating FPR can be an effective biomarker for identifying high-risk patients and choosing suitable therapeutics for early-stage CRC.

## Introduction

Colorectal cancer (CRC) is the second most common digestive malignancy and the fifth leading cause of cancer-related deaths in China [1], accounting for approximately 30% of all annually diagnosed CRC and disease-related deaths worldwide [2]. Due to no obvious clinical symptoms in the early-stage disease, the most clinically diagnosed patients are in advanced, leading to a poor prognosis [3]. Colonoscopic polypectomy and surgical resection are the primary methods that can radically treat benign colorectal polyp and stage I–II CRC, respectively [4]. Hence, detection and treatment of precancerous lesions and early-stage cancers can be highly effective in decreasing the morbidity and mortality caused by CRC.

Commonly, colorectal polyps can be histologically classified as adenomatous or non-neoplastic. Non-neoplastic polyps typically have no malignant potential, such as hyperplastic, hamartomatous, and inflammatory polyps. Neoplastic polyps, including colorectal tubular and tubulovillous adenomatous polyps, and serrated hyperplastic polyps are capable of developing adenocarcinomas through the classic adenoma-carcinoma pathway and serrated pathway, respectively [5]. However, the colonoscopic appearance of malignant colorectal polyps containing invasive CRC is not easily distinguishable from non-neoplastic and benign adenomatous polyps [6].

Accumulating evidence shows that chronic inflammation and genetic variation play key roles in colorectal carcinogenesis via premalignant polyps [7]. Colorectal precursor lesions commonly harbor inflammatory histologic characteristics, while inflammation-promoted DNA damage has been widely examined in cancer and precancerous lesions [8]. The microenvironmental inflammatory process stimulates angiogenesis, promotes cell proliferation, and inhibits apoptosis to encourage the process of polyps to CRC [9]. Our previous studies showed that circulating fibrinogen (Fib) to pre-albumin (pAlb) ratio (FPR) and albumin (Alb) to fibrinogen ratio (AFR) are two sensitive biomarkers reflecting host inflammation [10–13]. Circulating FPR was reported as a promising biomarker for diagnosis of CRC [10], and the two had better prognostic performances than the other inflammatory biomarkers for the localized non-small cell lung cancer and CRC, respectively [11, 14]. However, the differential diagnosis values of AFR and FPR in the subsets of colorectal polyps and early-stage CRC remain unknown. No study has reported their roles in identifying clinical high-relapse risk patients with early-stage CRC.

In our study, a discovery (155 patients with non-neoplastic polyps, 539 patients with colorectal adenomatous polyps, and 512 stage I–II CRC patients) and a validation (201 cases with benign colorectal polyps and 202 subjects with stage I–II CRC) cohort were enrolled. This study aimed to investigate: (1) the diagnostic efficacy of FPR and AFR in early-stage CRC and subsets of colorectal polyps and (2) the discriminating role of circulating FPR in identifying high-relapse risk patients with stage II CRC after the radical operation.

## Patients and methods

**Fig. 1:**
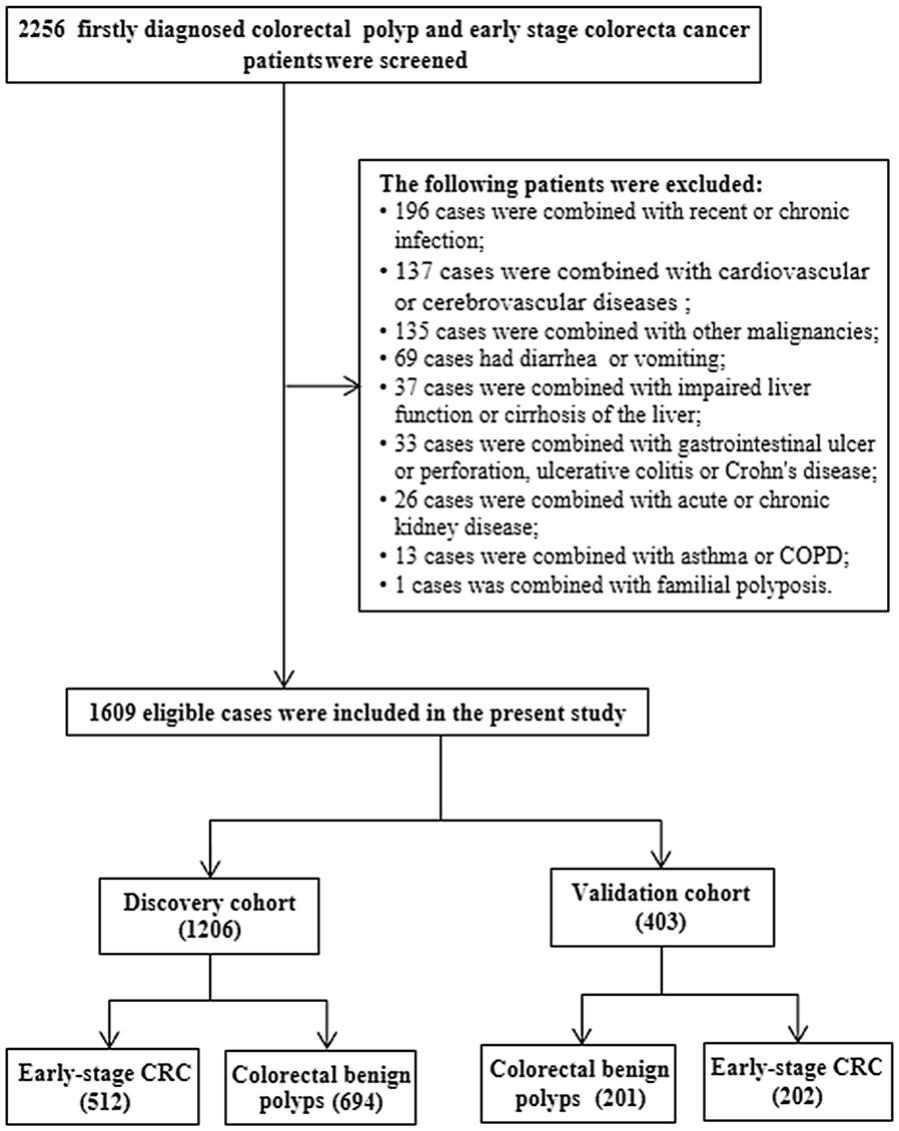
Screening and identifying flowchart of eligible patients in the present study

The ethics committee of the Second Affiliated Hospital of Nanchang University approved this study. All procedures were performed following the guidelines of the Declaration of Helsinki, and we obtained written informed consent from each participant. This study was carried out in a double blind experiment setting, Cui-Feng Xiong screened and identified the eligible patients from the hospital, Wei Chen collected the clinical samples and performed the follow-up, Yuanyuan Wang performed the laboratory detection. A flowchart for the selection of eligible participants is shown in Fig. 1. Firstly, we screened for newly diagnosed cases of colorectal polyps (January of 2013–December of 2019) and stage I–II CRC patients (January of 2013–October of 2018) in the hospital. The eligible patients were screened according to the following inclusion criteria: (a) clinical baseline, information, and the blood samples were provided; (b) they did not receive any treatment nor were administered non-steroidal anti-inflammatory drugs before the clinical diagnosis; (c) cases were confirmed by biopsy and pathological detection by two senior pathologists; and (d) all the early-stage CRC patients underwent the curative operation, and cancer resected margins were negative. Secondly, the following patients were excluded according to the following criteria: (a) loss of following-up in the first 6 months; (b) diarrhea, vomiting, and presence of diseases including inflammatory bowel disease, hereditary polyposis, other malignancies, and polyps from other organs; and (c) the patients harbored acute infection, autoimmune or chronic kidney disease, hematopathy, hepatopathy, or cardiovascular and cerebrovascular disease in the past month. Thirdly, all the eligible patients were divided into a discovery (before 2017) and a validation cohort (after 2017), and we selected the enrolled time to classify the overall population to keep unbiased segregation of/ the two cohorts.

We collected clinical baseline and pathological characteristics from each patient. The laboratory-detected sample, 2-mL plasma and serum, were collected at the time of admission, which was earlier than any other treatments at the hospital. Plasma fibrinogen (Fib) was detected by the Clauss assay using a SYSMEX CA-7000 machine (Sysmex, Tokyo, Japan). Bromocresol green staining method and immunological turbidimetry assays were used to measure the concentrations of serum albumin (Alb) and pre-albumin (pAlb) using the OLYMPUS AU5400 (Beckman Coulter, Tokyo, Japan), respectively. A chemiluminescence immunoassay was used to detect serum carcinoembryonic antigen (CEA) and carbohydrate antigen 19-9 (CA19-9) using a Siemens ADVIA Centaur XP machine (Siemens, Erlangen, Germany). All the detection was completed within two hours after sample collection. The inter- and intra-batch coefficients of these detections were less than 7.5%. We calculated the Alb-to-Fib ratio (AFR = Alb/Fib) and the Fib-to-pAlb ratio (FPR = Fib*1000/pAlb) based on the results of detection.

Radical operation is typical to treat stage I-II CRC, and patients with stage I disease do not need adjuvant chemotherapy (CT) after surgery. However, adjuvant CT is necessary for stage II surgical patients with high-relapse risk. Clinical characteristics such as poor histological differentiation (G3-4), T4 stage, vascular lymphatic infiltration, preoperative intestinal obstruction, or intestinal perforation, and the number of lymph nodes detected in surgical specimens < 12, are used to identify stage II CRC patients with clinical high-relapse risk [15]. In this study, we classified the stage II cases into clinical high- and low-risk patients (HR and LR) in accordance with the criteria. A 3-year following-up performed every 3 months in the 1st year and every 6 months in the 2nd and 3rd year was conducted in the early-stage CRC subgroup. Recurrence-free survival (RFS) was the primary outcome in the present study and was measured from the time of curative resection to the time of disease recurrence or the set deadline. The deadline was set in June of 2021. Overall survival (OS) was defined as the time from surgery to death or the deadline of the study, whichever was earlier. In the follow-up period, patients who were detected with significantly elevated (> 2-fold) CEA or CA19-9, apparent recurrence imaging features, or colonoscopy observation were considered to have recurrence or distal metastasis of the disease.

The prognostic cut-off values of FPR within stage I and II CRC were 14.0 and 16.5, respectively, as reported in our previous study [16]. Binary and continuous variables were summarized as numbers and frequencies and medians and quartiles, respectively. Comparisons were analyzed using the Chi-square test, Fisher’s exact test, Kolmogorov–Smirnov, and Mann–Whitney U tests. The survival differences between the comparisons were compared using the Kaplan–Meier curve (log-rank test). Time-dependent receiver operating characteristic (ROC) curves, area under the curve (AUC), sensitivity (Sen), and specificity (Spe) were selected to evaluate the predicted efficacy on the 3-years RFS and OS. SPSS. 22.0 (IBM Corp, Armonk, NY, USA), R 3.5.1 (Institute for Statistics and Mathematics, Vienna, Austria) with packages of “tdROC”, and GraphPad Prism 8.2.1 (GraphPad Software Inc, San Diego, USA) were used for the statistical analyses, and *p* < 0.05 (two-sided) was recognized as statistically significant.

## Results

According to the inclusion and exclusion criteria, 2256 patients were enrolled and screened to identify eligible patients. As a result, 155 cases of colorectal non-neoplastic polyps (88 inflammatory and 67 hyperplastic polyps patients), 539 cases of colorectal adenomatous polyps, and 512 early-stage CRC patients (110 stage I patients and 402 stage II cases) were enrolled as eligible cases in the discovery cohort. The validation cohort consisted of 201 patients with benign colorectal polyps (colorectal non-neoplastic and adenomatous polyps) and 202 patients with stage I–II CRC (Fig. 1). The characteristics of the patients are summarized in Table 1. Significant sex and age distribution differences were observed between the benign colorectal polyps and early-stage CRC groups in the discovery cohort (all *p* < 0.01).

**Table 1:** The baseline and clinicopathological characteristics of eligible patients in the discovery and validation cohorts

Variables		Discovery cohort			Validation cohort		
		Colorectal benign polyps (694)	Early-stage colorectal cancer (512)	*p* value	Colorectal benign polyps (201)	Early-stage colorectal cancer (202)	*p* value
		N(%)	N(%)		N(%)	N(%)	
Gender	Male	433(62.39%)	312(60.94%)	< 0.001	139(69.15%)	115(56.93%)	0.011
	Female	261(37.61%)	200(39.06%)		62(30.85%)	87(43.07%)	
Age	< 60	410(59.08%)	238(46.48%)	< 0.001	108(53.73%)	110(54.46%)	0.884
	≥ 60	284(40.92%)	274(53.52%)		93(46.27%)	92(45.54%)	
Smoking	Yes	152(21.90%)	100(19.53%)	0.317	31(15.42%)	32(15.84%)	0.908
	No	542(78.10%)	412(80.47%)		170(84.58%)	170(84.16%)	
Drinking	Yes	114(16.43%)	77(15.04%)	0.514	30(14.93%)	27(13.37%)	0.653
	No	580(83.57%)	435(84.96%)		171(85.07%)	175(86.63%)	
Diabetes	Yes	46(6.63%)	37(7.23%)	0.685	16(7.96%)	14(6.93%)	0.694
	No	648(93.37%)	475(92.77%)		185(92.04%)	188(93.07%)	
Hypertension	Yes	136(19.60%)	101(19.73%)	0.955	36(17.91%)	36(17.82%)	0.981
	No	558(80.40%)	411 (80.27%)		165(82.09%)	166(82.18%)	
TNM stage	I	–	110(21.48%)		–	49(24.26%)	
	II	–	402(78.52%)		–	153(75.74%)	
T stage	T1–2	–	110(21.48%)		–	52(25.74%)	
	T3–4	–	402(78.52%)		–	150(74.26%)	
Differentiation	G1–2	–	488(95.31%)		–	–	
	G3–4	–	24(4.69%)		–	–	
Radical surgery	Yes	–	512(100%)		–	202(100%)	
Chemotherapy	Yes	–	382(74.61%)		–	118(58.42%)	
	No	–	130(25.39%)		–	84(41.58%)	
CEA (> 5 ng/mL)	< 5	676(97.41%)	389(75.98%)	< 0.001	198(98.51%)	145(71.78%)	< 0.001
	≥ 5	18(2.59%)	123(24.02%)		3(1.49%)	57(28.22%)	
CA19-9 (> 37U/mL)	< 37	657(94.67%)	442(86.33%)	< 0.001	197(98.01%)	176(87.13%)	< 0.001
	≥ 37	37(5.33%)	70(13.67%)		4(1.99%)	26(12.87%)	
Fib (g/L)		2.45(2.10–2.81)	3.05(2.55–3.60)	< 0.001	2.51(2.18–2.99)	3.07(2.54–3.83)	< 0.001
Alb(g/L)		42.00(39.74–44.10)	40.68(38.50–42.64)	< 0.001	41.73(39.72–44.13)	40.72(37.79–43.63)	< 0.001
preAlb (mg/L)		261.72(218.74–305.10)	210.73(168.60–259.22)	< 0.001	246.26(197.07–287.97)	181.40(135.45–229.21)	< 0.001
AFR		17.11(14.87–20.02)	13.26(10.98–15.86)	< 0.001	16.37(14.37–19.39)	13.40(10.09–16.13)	< 0.001
FPR		9.54(7.68–11.72)	14.50(10.87–19.11)	< 0.001	10.72(8.34–13.75)	17.05(12.12–26.41)	< 0.001

FPR = Fib/pAlb × 1000; AFR = Alb/Fib; distribution differences of gender, age, smoking, drinking, diabetes, hypertension, CEA, CA19-9 between the groups were tested by Chi-square test; Fib, Alb, pAlb, AFR, FPR differences between groups were tested by rank-sum test

*CRC* colorectal cancer; benign colorectal polyps include colorectal non-neoplastic and adenomatous polyps; *Fib* Fibrinogen; *Alb* albumin; *pAlb* pre-albumin

There was only a sex distribution difference in the validation cohort (*p* = 0.011). All eligible patients underwent endoscopic resection or curative surgical operation, and 382 and 118 CRC patients received CT after surgery in the discovery and validation cohorts, respectively. Compared to the non-neoplastic and adenoma polyp subgroups, circulating Fib and FPR were significantly higher in the CRC subgroup (all *p* < 0.01), conversely, Alb, pAlb, and AFR were low in early-stage CRC patients compared to colorectal benign polyps cases in the two cohorts (all *p* < 0.01).

**Fig. 2:**
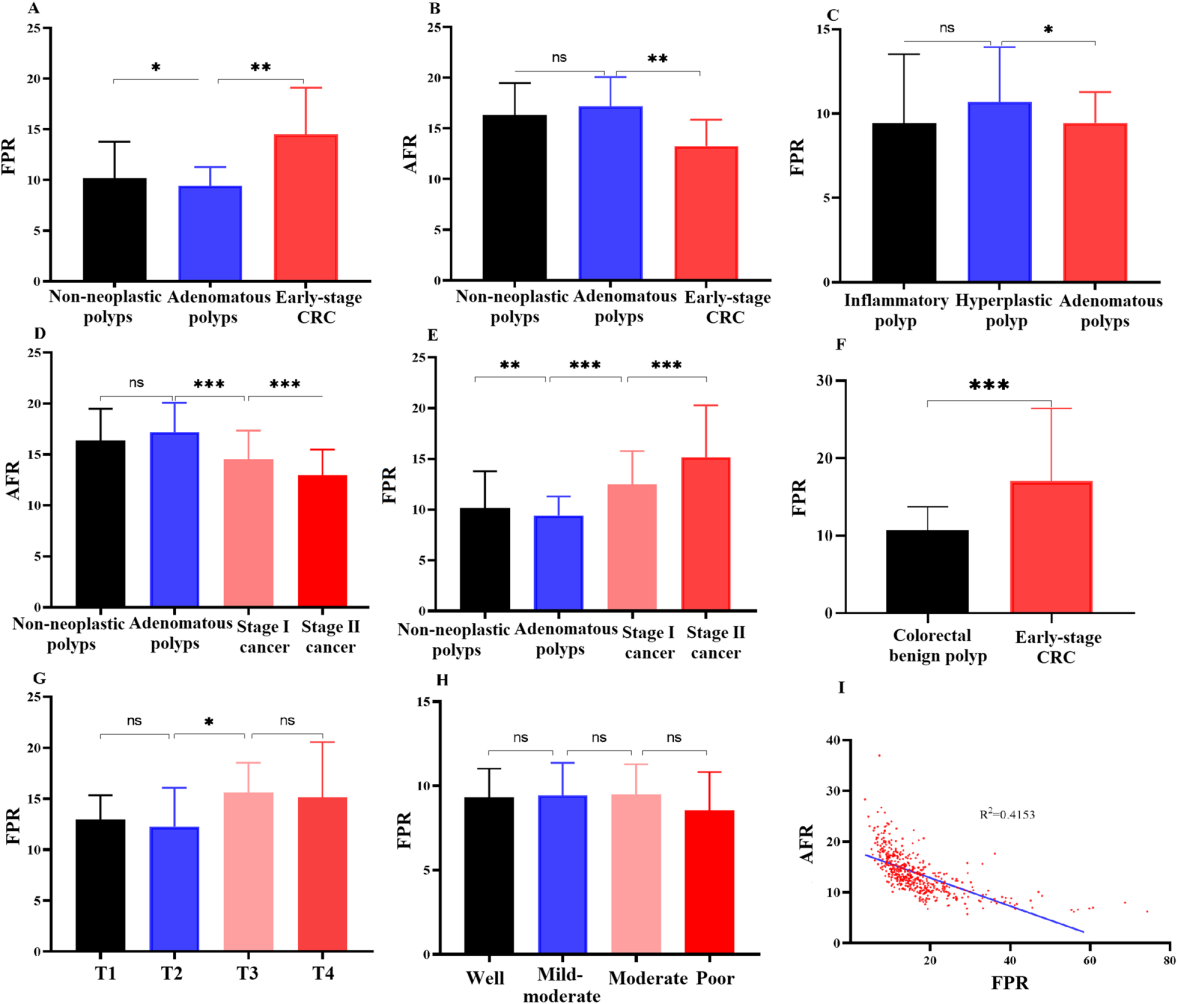
Circulating FPR (**A**, **C**, **E**–**H**) and AFR (**B**, **D**) comparisons in subsets of colorectal polyps, stage I–II CRC patients, and subgroups stratified by cancer invasion and histological differentiation, as well as the relationship of the two ratios in the overall population (**I**). *p < 0.05; **p < 0.01; ***p < 0.001; *ns* no significance

Among the colorectal non-neoplastic and adenomatous polyp subgroups, there were differences observed in circulating FPR (*p* < 0.05) and AFR (*p* > 0.05) in the discovery cohort (Fig. 2A, B). Circulating FPR was significantly lower in patients with adenomatous polyps than in those with hyperplastic polyps (*p* < 0.05) (Fig. 2C). However, there was no difference in FPR between the inflammatory and hyperplastic polyp subgroups (Fig. 2C). The AUCs of FPR, AFR, CEA, and CA19-9 for discriminating colorectal non-neoplastic and adenomatous polyps were 0.576, 0.549, 0.507, and 0.528, respectively (Table 2). Circulating AFR gradually reduced from colorectal adenoma to stage I and stage II CRC subgroups. A significant difference in AFR was observed in patients with benign colorectal benign polyps and early-stage CRC (Fig. 2D). In contrast, circulating FPR was gradually increased in these subgroups (Fig. 2E), and a significantly higher FPR was also observed in early-stage CRC than in the benign colorectal polyps group (Fig. 2F). In early-stage CRC, a considerably higher FPR was observed in the T3–4 subgroup than that in the T1–2 patients; however, no difference in FPR was observed in the comparisons of T1 vs. T2, T3 vs. T4 (Fig. 2G) or subgroups stratified by histological differentiation (Fig. 2H).

**Table 2:** The diagnostic efficacy of preoperative FPR, AFR, CEA, CA19-9, and FPR combined with CEA and CA19-9 in patients with colorectal non-neoplastic polyps, adenomas, and early-stage colorectal cancer in discovery and validation cohorts and overall population

Comparison		Biomarkers	Cut-off value	AUC	Sensitivity (%)	Specificity (%)	PPV (%)	NPV (%)	Youden’s index
Discovery cohort	Non-neoplastic polyps vs. adnomas	FPR	6.17	0.576	91.10	14.20	78.70	31.40	0.053
		AFR	22.69	0.549	12.90	87.10	77.78	77.65	0.027
		CEA	1.245	0.507	57.80	48.10	23.59	80.48	0.059
		CA19-9	8.165	0.528	70.40	38.80	80.60	26.63	0.095
	Adnomas vs. early-stage CRC	FPR	11.73	0.818	70.60	79.70	80.37	69.67	0.503
		AFR	14.90	0.767	76.10	66.90	66.11	76.74	0.430
		CEA	1.895	0.711	63.80	68.30	70.38	61.58	0.321
		CA19-9	16.895	0.577	41.60	73.30	64.67	51.60	0.149
		CEA+FPR	0.63	0.858	67.10	90.90	89.70	70.11	0.580
		CEA+CA19-9+FPR	0.62	0.858	67.10	90.70	89.46	70.06	0.578
	Colorectal benign polyps vs. early-stage CRC	FPR	12.47	0.792	65.10	81.30	72.25	71.62	0.464
		AFR	14.90	0.754	75.50	33.10	54.98	55.44	0.424
		CEA	1.875	0.711	63.80	69.40	65.83	67.51	0.332
		CA19-9	16.86	0.582	41.80	73.60	59.88	57.86	0.154
		CEA+FPR	0.50	0.835	68.30	83.40	79.11	74.04	0.525
		CEA+CA19-9+FPR	0.50	0.835	68.60	83.90	79.72	74.30	0.525
Validation cohort	Colorectal benign polyps vs. early-stage CRC	FPR	12.47	0.759	72.30	68.20	69.52	79.98	0.405
		AFR	14.90	0.703	70.60	65.30	67.14	68.95	0.359
		CEA	1.875	0.702	58.90	68.20	65.03	62.27	0.271
		CA19-9	16.86	0.579	43.60	73.10	61.97	56.32	0.167
		CEA+FPR	0.50	0.823	61.90	83.60	79.11	68.57	0.455
		CEA+CA19-9+FPR	0.50	0.823	61.90	83.60	79.11	68.57	0.455
Overall population	Colorectal benign polyps vs. early-stage CRC	FPR	12.47	0.780	67.20	77.80	69.08	76.27	0.450
		AFR	14.90	0.742	74.00	66.40	60.97	75.70	0.404
		CEA	1.875	0.709	62.40	68.40	59.29	71.12	0.308
		CA19-9	16.86	0.580	42.20	73.50	29.75	38.33	0.157
		CEA+FPR	0.50	0.829	63.20	86.00	76.96	76.81	0.492
		CEA+CA19-9+FPR	0.50	0.828	64.00	85.70	76.05	76.38	0.497

FPR = Fib/pAlb × 1000; AFR = Alb/Fib

*CRC* colorectal cancer; benign colorectal polyps include colorectal non-neoplastic and adenomatous polyps; *Fib* Fibrinogen; *Alb* albumin; *pAlb* pre-albumin; *PPV* positive predictive value; *NPV* negative predictive value; *AUC* area under curve

**Fig. 3:**
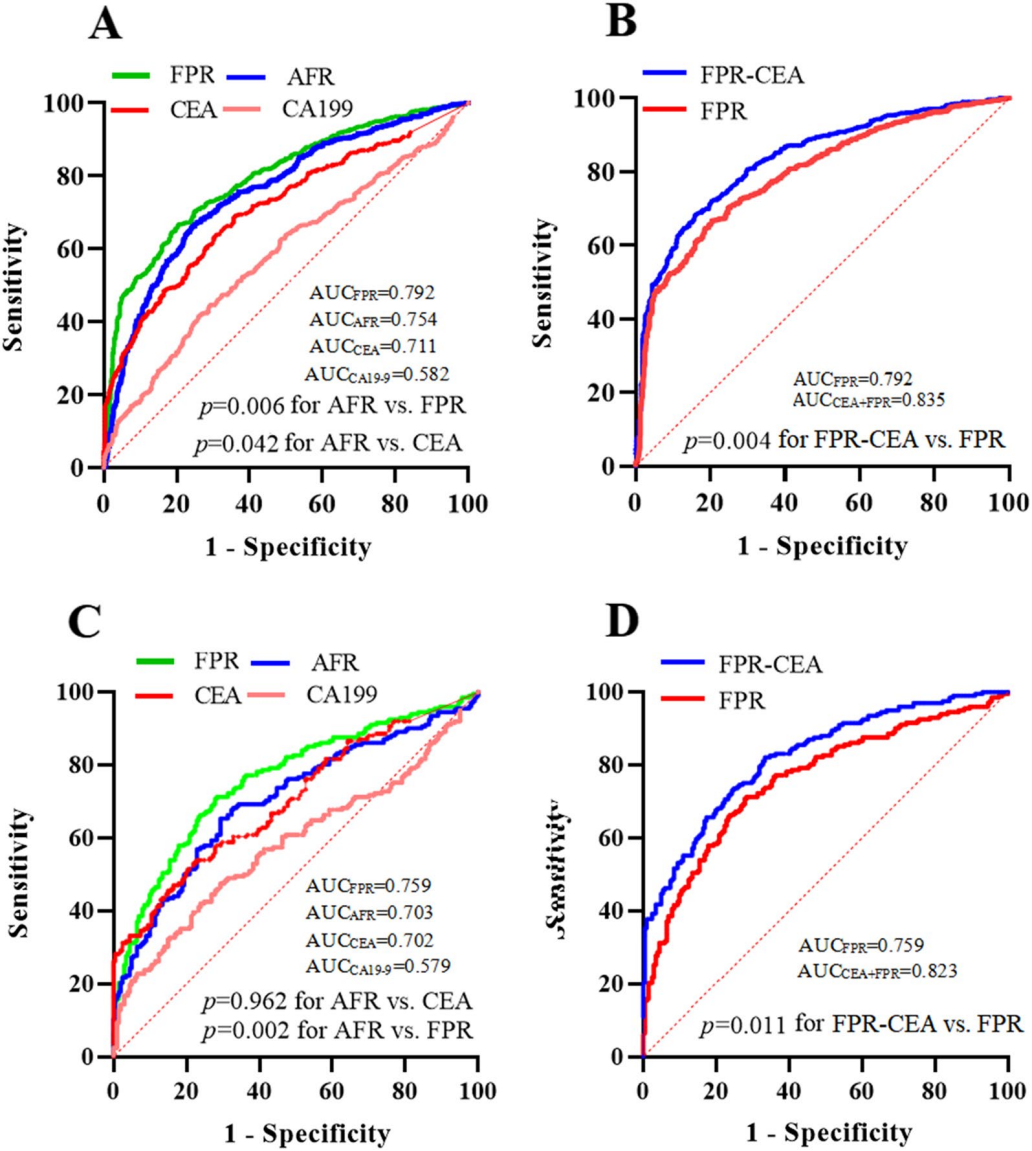
Receiver operation characteristic curve analysis of FPR, AFR, CEA, CA19-9, combined FPR-CEA, and FPR-CEA-CA19-9 in early-stage CRC and benign colorectal polyps in the discovery (**A**, **B**) and validation (**C**, **D**) cohort

In the discovery cohort, the AUCs of FPR, AFR, CEA, and CA19-9 were 0.818, 0.767, 0.711, and 0.577 for the differential diagnosis of early-stage CRC and colorectal adenomas polyps, respectively (Table 2). The Sen and Spe of FPR (cut-off = 11.73, Sen = 70.60%, Spe = 79.70%) and AFR (cut-off = 14.90, Sen = 76.10%, Spe = 66.90%) were better than those of CEA and CA19-9, respectively (Table 2). In discriminating CRC from benign colorectal polyps, the AUCs, Sen, and Spe were 0.792, 65.10%, and 81.30%, for FPR, respectively, and 0.754, 75.50%, and 33.10% for AFR, respectively, and their AUCs were superior to CEA and CA19-9, respectively (Fig. 3A, Table 2). Circulating AFR was negatively correlated with FPR in the overall population (Fig. 2I). We selected FPR, CEA, and CA19-9 to evaluate the combined diagnostic efficacy in discriminating colorectal adenoma polyps and early-stage CRC. We observed that AUCs, Sen, and Spe of the combined CEA-FPR were 0.858, 67.10%, and 90.90%, respectively, which was similar to the combined CEA-CA19-9-FPR (Table 2). In benign colorectal polyps and early-stage CRC subgroups, the AUCs of the combined CEA-FPR and CEA-FPR-CA19-9 were 0.835 and 0.835, respectively. Their sensitivities were 68.30% and 68.60%, with specificity of 83.40% and 83.90%, respectively (Fig. 3B, Table 2).

In the validation cohort, the AUC of FPR was significantly higher than that of AFR, CEA, and CA19-9 in diagnosing early-stage CRC from colorectal benign polyps (all *p* < 0.01) (Fig. 3C). The AUCs of FPR-CEA and FPR-CEA-CA19-9 were similar and were effectively improved compared to the single FPR (0.823 vs. 0.759, *p* < 0.01) (Fig. 3D, Table 2). AUC, Sen, Spe, positive predictive value, negative predictive value, and Youden’s index of FPR were 0.780, 67.20%, 77.80%, 69.08%, 76.27%, and 0.450 for the diagnosis of early-stage CRC and colorectal benign polyps, respectively, which were better than the other single biomarkers in the overall population (Table 2). The diagnostic efficacy of combined FPR and CEA was similar to that of FPR-CEA-CA19-9; however, the AUCs of combined FPR-CEA (0.829 vs. 0.780, *p* = 0.011) and FPR-CEA-CA19-9 were significantly higher than those of FPR in the overall population (Table 2).

**Fig. 4:**
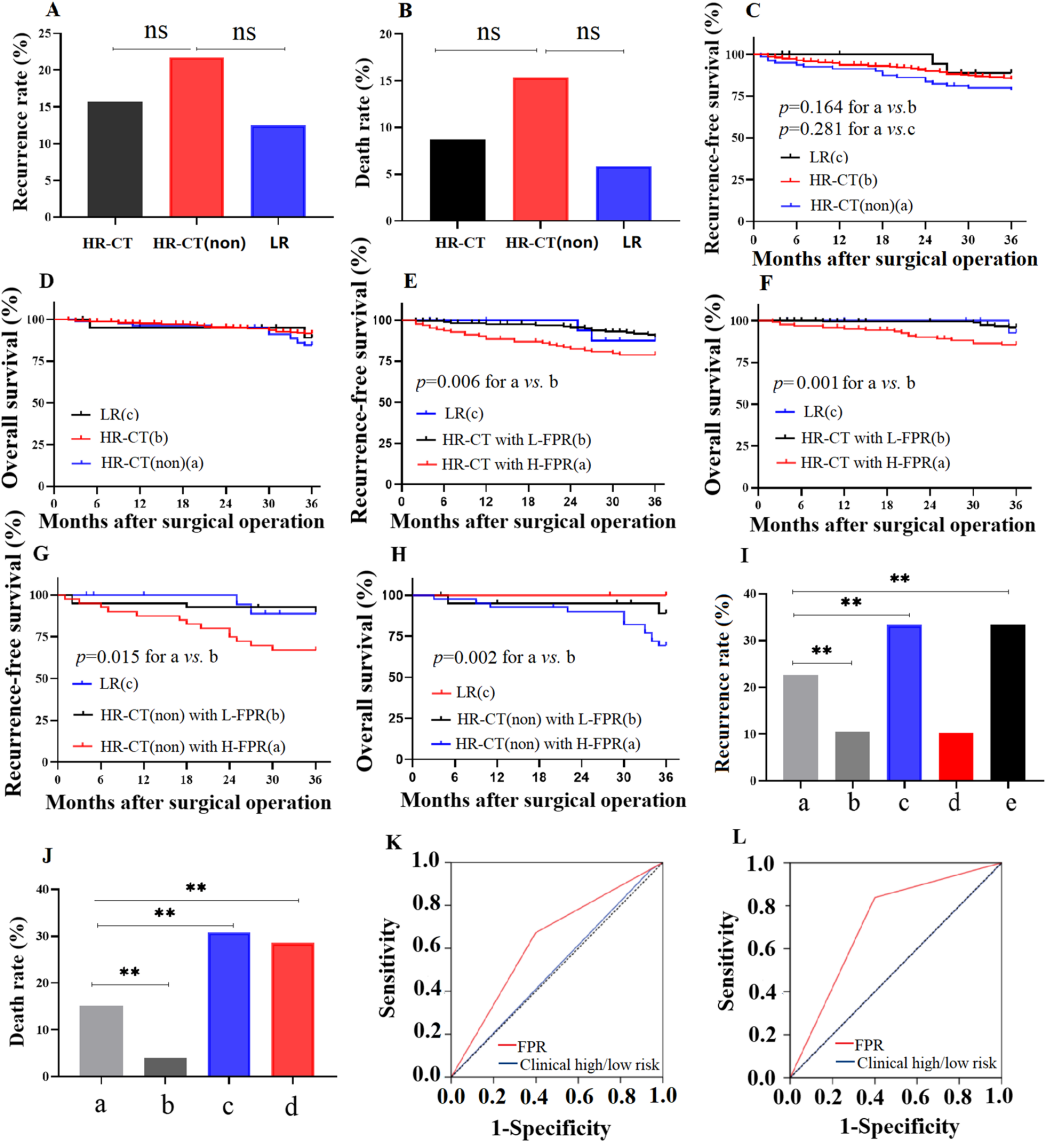
Prognosis and predicted efficacy of FPR and clinical characteristics in identifying a recurrence of stage II CRC patients. Recurrence (**A**) and death rate (**B**) in clinical low-risk (LR) and high-risk (HR) patients with or without chemotherapy (CT); **C**, **D** Kaplan–Meier (K–M) curve of survival in HR-CT, HR-CT(non), LR subgroups; **E**, **F** K–M curve of survival in HR-CT patients with high- and low-FPR and LR subgroup; **G**, **H** K–M curve of survival in HR-CT(non) patients with high- and low-FPR and LR subgroup; **I**, **J** recurrence and death rate in HR-CT group with H-(a) and L-(b) FPR, LR group with H-FPR (c), HR-CT(non) with L-(d) and H-(e) FPR; **K**, **L** time-dependent ROC of FPR and common clinical characteristics in predicting the 3 years RFS and OS; **p < 0.01; *ns* no significance

According to the criteria of clinical high/low-risk patients, we divided the patients into clinical HR (572 cases) and LR (32 cases) groups. Although the recurrence rate in the non-CT-treated patients was higher than in CT-treated patients in clinical HR (21.79% vs. 15.73%, *p* = 0.211) and LR (21.79% vs. 12.50%, *p* = 0.512) subgroups, no statistical difference was observed between them (Fig. 4A). Similarly, there was also no difference in the comparison of death rates between CT-treated and non-CT-treated clinical HR [HR-CT, HR-CT(non)] patients and LR cases (15.38% vs. 8.75% vs. 5.88%, *p* = 0.234) (Fig. 4B). Furthermore, no survival (RFS and OS) differences were observed in the two subgroups (Fig. 4C, D).

According to the cut-off value of FPR, stage II patients were classified into high-FPR (H-FPR) and low-FPR (L-FPR) subgroups. RFS and OS were shorter in H-FPR patients than in L-FPR patients in clinical CT-treated (*p*_log-rank_ = 0.006 for RFS, *p*_log-rank_ = 0.001 for OS) and non-CT-treated (*p*_log-rank_ = 0.015 for RFS, *p*_log-rank_ = 0.002 for OS) HR subgroups with the stage II disease (Fig. 4E–H). However, no survival difference was observed between the clinical LR subgroup and HR patients with L-FPR regardless of treatment with CT (Fig. 4E–H).

Clinical LR patients and non-CT-treated clinical HR patients with H-FPR harbored the highest recurrence rate (33.33%), while L-FPR patients with clinical HR risk had the lowest recurrence rate regardless of treatment with CT (10.26% for non-CT treated patients; 10.53% for CT treated patients) (Fig. 4I). In the clinical HR subgroup, the recurrence rate in H-FPR patients treated with CT was significantly lower than that in the non-CT-treated patients (22.61% vs. 33.33%, *p* < 0.001) but was considerably higher than that in L-FPR patients treated with CT (22.61% vs. 10.53%, *p* < 0.001) (Fig. 4I). The lowest and highest death rates were observed in CT-treated clinical HR patients with L-FPR (3.97%) and H-FPR patients with clinical LR (28.57%) and CT-treated H-FPR patients with clinical HR (30.77%) (Fig. 4J). The death rate of CT-treated clinical HR patients with H-FPR was significantly lower than that of non-CT-treated clinical HR patients with H-FPR (15.18% vs. 30.77%, *p* < 0.001) and clinical LR patients with H-FPR (15.18% vs. 28.57%, *p* < 0.001) (Fig. 4J). Additionally, the efficacy of FPR and common clinical characteristics predicted that the 3-years RFS and OS were 0.637 and 0.511, and 0.719 and 0.501, respectively. The AUC of FPR was significantly higher than clinical characteristics in predicting the prognosis (Fig. 4K, L).

**Fig. 5:**
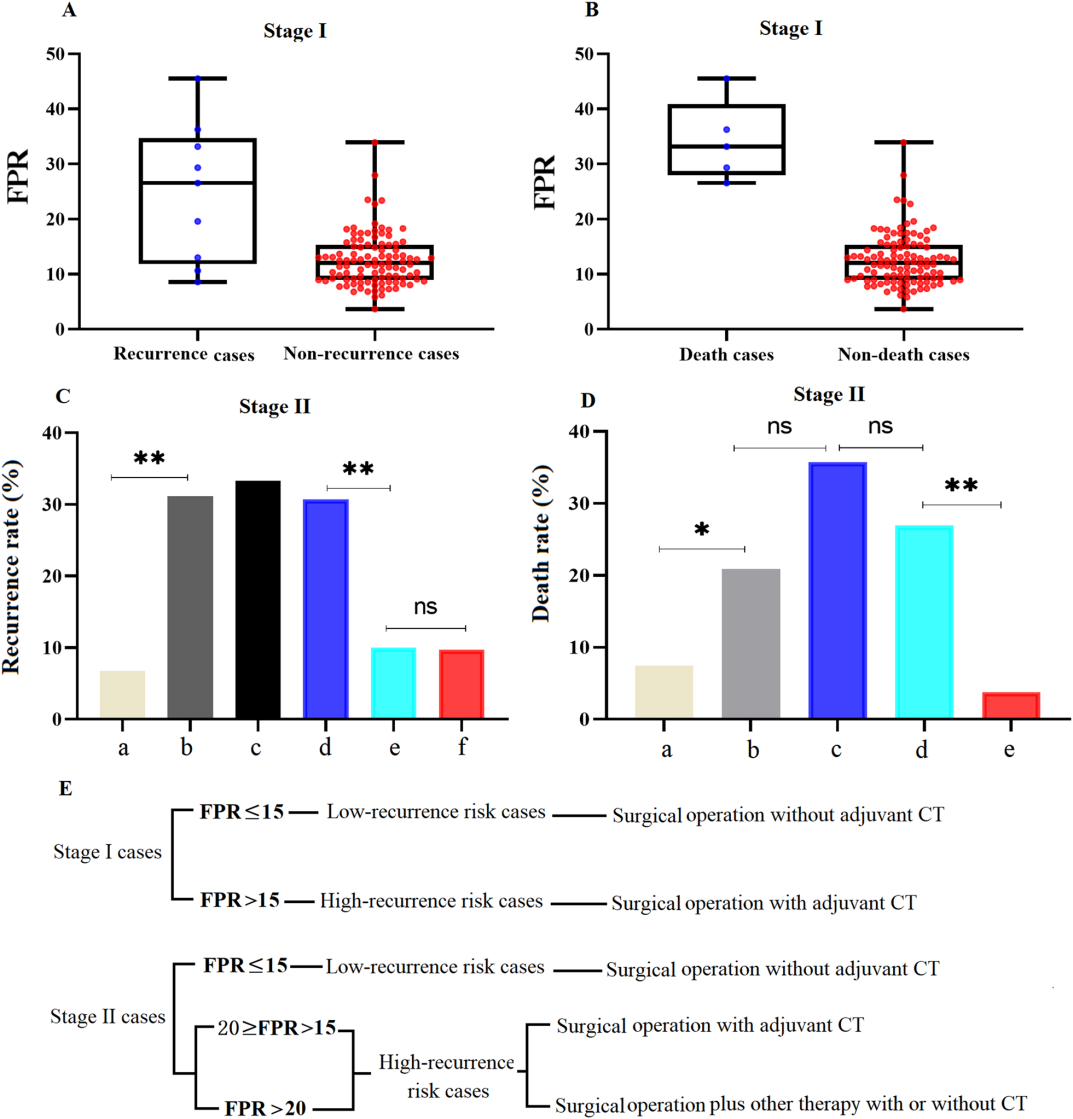
Circulating FPR, chemotherapy, and prognosis of early-stage CRC. **A**, **B** FPR comparisons between stage I CRC patients with or without recurrence/death; **C**, **D** Recurrence and death rates of CT- and non-CT-treated stage II CRC patients with 20.0 ≥ FPR > 15 (a: CT-treated patients; c: non-CT-treated patients), or FPR > 20 (b: CT-treated patients; d: non-CT-treated patients) and FPR ≤ 15 (e: CT-treated patients; f: non-CT-treated patients); **E** The therapeutic selection according to circulating FPR. **p < 0.01; *ns* no significance

In patients with stage I CRC, the recurrence rate was only 3.85% in patients with L-FPR (≤ 15), and no deaths were observed in the two subgroups. In H-FPR (> 15) patients, recurrence (18.18%) and death (15.15%) were observed at the 3-year follow-up. A significantly higher FPR was also observed in recurrence and death cases compared to non-recurrence and non-death cases (all *p* < 0.01) in stage I CRC patients, respectively (Fig. 5A, B). In H-FPR stage II CRC patients, recurrence and death rates of CT-treated patients with FPR > 20 and non-CT treated patients with 20 ≥ FPR > 16.5 or FPR ≥ 20 were 31.16% and 21.88%, 33.30% and 35.71%, 30.70% and 26.82%, respectively, and no difference was observed between them. However, the recurrence and death rates of CT-treated patients with 20 ≥ FPR > 15, L-FPR patients (≤ 15) with or without CT were 6.77% and 7.41%, 10.00% and 3.77%, and 9.76%, respectively, and the rates were significantly lower than those of CT-treated patients with FPR > 20, and non-CT treated patients with 20 ≥ FPR > 15 or FPR > 20 (Fig. 5C, D).

## Discussion

Cancer-related inflammatory biomarkers may aid in identifying the early-stage disease, discriminating clinical high-risk stage II patients, and guiding therapeutics. This study found significantly high FPR and low AFR in early-stage CRC compared to subsets of colorectal polyps. Preoperative FPR was superior to AFR and is considered a common tumor biomarker that may be used to diagnose early-stage CRC from benign colorectal polyps in the discovery and validation cohorts and the overall population. Preoperative FPR combined with CEA could effectively distinguish early-stage cancer from benign colorectal polyps, with an AUC, Sen, and Spe of 0.835, 68.30%, and 83.40% in the discovery cohort, 0.823, 61.90%, and 83.60% in the validation cohort, and 0.829, 63.20%, and 86.00% in the overall population, respectively. Moreover, circulating FPR identified stage II patients with a high relapse risk after surgical operation, and its predicted efficacy was superior to that of common clinical characteristics. Additionally, preoperative FPR could help clinicians choose suitable therapeutics for stage I and II disease patients.

It is well known that most CRCs develop from colorectal adenomatous or serrated polyps [17, 18]. Screening, identification, and removal of the precancerous lesion and early-stage CRC can effectively reduce incidence and mortality[19]. The fecal immunochemical test (FIT) is the preferred and most used method to screen the early-stage CRC; however, its sensitivity needs further improvement, particularly in adenoma cases [20]. Combined multitarget stool or serum DNA methylation site tests and FIT can improve sensitivity [21–23]. The cost of testing restricts its wide use in clinics, particularly in primary medical units. Although colonoscopy and sigmoidoscopy are significantly advantageous, most individuals are unwilling to undergo the procedures due to their invasive characteristics, especially during healthy check-ups [24].

Inflammation induces carcinogenic mutagenesis and regulates carcinogenesis of CRC [25–27]. Common inflammatory ratios such as neutrophil to lymphocyte ratio, platelet to lymphocyte ratio, and lymphocyte to monocyte ratio show moderate diagnostic efficacies in distinguishing glioma, lung cancer, and healthy subjects, respectively [28, 29]. Our previous study also found that circulating FPR was superior to AFR and NLR in diagnosing stage I–IV CRC in healthy individuals [30]. In this study, the diagnostic AUCs of AFR and FPR were less than 0.60 in the diagnosis of colorectal non-neoplastic and adenomatous polyps, indicating that the two ratios could not differentially diagnose the subsets of colorectal polyps. Emir et al. also observed no significant differences in NLR and PLR in colorectal polyp cases and healthy individuals [31]. AFR and FPR were gradually decreased and increased in colorectal adenoma, stage I, and stage II CRC, respectively. Their diagnostic efficacies were high (up to 0.75), suggesting that the two ratios could effectively distinguish early-stage CRC from adenoma and other subsets of colorectal polyps. The AUC of the combined CEA-FPR was equal to that of CEA-FPR-CA19-9, and their sensitivity and specificity were higher than those of the single biomarkers, showing that the combined CEA-FPR was superior to FPR or AFR in identifying early-stage CRC from benign colorectal polyps.

According to the CRC guidelines, most stage II cases are not recommended to receive adjuvant CT after surgery, except for clinical HR patients [15]. In this study, we used the following clinical characteristics: poor histological differentiation (G3–4), T4 stage, vascular lymphatic infiltration, preoperative intestinal obstruction or intestinal perforation, and the number of lymph nodes detected in surgical specimens < 12 to classify patients with stage II CRC into two subgroups with clinical HR and LR. In CT-treated or non-CT-treated patients, we found clinical prognosis and recurrence rate to be the same between clinical HR and LR cases. No difference in the rate was observed in each clinical HR and LR group with or without CT. Moreover, the predicted time-dependent AUCs of clinical characteristics were 0.511 and 0.501 for 3-year RFS and OS, respectively. These findings demonstrated that the typical clinical features could not effectively distinguish between clinical HR and LR patients and that clinically HR patients could not benefit from adjuvant CT after surgical operation.

Different treatment efficacies in chemotherapeutic drug responses are related to the different grades of chronic inflammation in patients [32, 33]. The lowest recurrence rate was found in L-FPR stage II clinical HR patients regardless of treatment with CT. In contrast, the highest recurrence rate was found in non-CT-treated H-FPR patients irrespective of clinical HR or LR, and the rate was approximately three times higher compared to patients treated with CT. Moreover, the rate was significantly decreased in H-FPR clinical HR patients receiving adjuvant CT compared to non-CT-treated patients. A similar result was also found in these patients concerning the death rate. L-FPR patients with stage II CRC had better survival than H-FPR patients in clinical HR and LR subgroups with or without CT. These results illustrated that H-FPR patients could benefit from CT, and L-FPR cases might not have received CT after surgery. Additionally, the predicted efficacy of FPR was much higher than the clinical characteristics for predicting the 3-year outcomes, indicating that FPR was superior to typical clinical features in identifying HR patients who can benefit from CT.

Our previous studies showed that high-grade chronic inflammation could attenuate chemosensitivity or even chemoresistance. Patients with low-grade FPR (≤ 15) showed complete response to CT; however, median-grade FPR (20 ≥ FPR > 15) and high-grade FPR (> 20) implied chemosensitivity and chemoresistance in CRC patients [16]. This study found that CT-treated FPR > 20 patients harbored the highest recurrence and death rates. Similar highest recurrence and death rates were observed in non-CT-treated patients with FPR > 20, indicating that the cases with FPR > 20 might not benefit from CT, and that these patients may be treated with single-targeted therapy or onco-immunotherapy, combined CT after the surgery [34–36]. Moreover, the recurrence and death rates in non-CT treated patients with 20 ≥ FPR > 15 were approximately five times higher than those of the CT-treated cases, suggesting that patients with 20 ≥ FPR > 15 were suitable for receiving CT and could significantly benefit from the treatment. Additionally, no significant difference in recurrence and death rates was observed between L-FPR patients (FPR ≤ 15) with or without CT, indicating that these patients may undergo surgical operation only and may not need to receive adjuvant CT after curative resection (Fig. 5E).

To the best of our knowledge, this study is the first study to investigate the role of FPR in distinguishing early-stage cancer from benign polyps and identifying patients with clinical high-relapse risk. Although preoperative FPR can effectively diagnose stage I–II CRC from benign colorectal polyps, it is not a specific biomarker for CRC. Therefore, FPR combined with CEA could improve the diagnostic efficacy for early-stage CRC. We also found that circulating FPR was superior to common clinical characteristics in identifying high-relapse risk patients who need to receive adjuvant CT. However, this study was only performed in a single-center, and a multi-center study with a large sample size should validate the findings.

## Conclusion

Circulating FPR is an effective biomarker to distinguish early-stage CRC from subsets of colorectal polyps, identify high-risk stage II CRC patients, and choose suitable therapeutics. FPR combined with CEA can improve the efficacy and sensitivity of diagnosing early-stage CRC.

## Data Availability

All data generated or analyzed during this study are included in this published article.

## Abbreviations

CRC: Colorectal cancer
FPR: Fibrinogen to pre-albumin ratio
AFR: Albumin to fibrinogen ratio
ROC: Receiver operating characteristic curve
AUC: Area under the curve
CEA: Carcinoembryonic antigen
CT: Chemotherapy
RFS: Recurrence-free survival
OS: Overall survival
L-FPR: Low-FPR
H-FPR: High-FPR
Fib: Fibrinogen
Alb: Albumin
pAlb: Pre-albumin
HR: Hazard
CI: Confidence interval
Sen: Sensitivity
Spe: Specificity
